# Small ncRNA Expression-Profiling of Blood from Hemophilia A Patients Identifies miR-1246 as a Potential Regulator of *Factor 8* Gene

**DOI:** 10.1371/journal.pone.0132433

**Published:** 2015-07-15

**Authors:** Tewarit Sarachana, Neetu Dahiya, Vijaya L. Simhadri, Gouri Shankar Pandey, Surbhi Saini, Christine Guelcher, Michael F. Guerrera, Chava Kimchi-Sarfaty, Zuben E. Sauna, Chintamani D. Atreya

**Affiliations:** 1 Laboratory of Cellular Hematology, Division of Hematology Research and Review, Center for Biologics Evaluation and Research, Food and Drug Administration, Silver Spring, Maryland 20993, United States of America; 2 Department of Clinical Chemistry, Faculty of Allied Health Sciences, Chulalongkorn University, Bangkok, 10330, Thailand; 3 Laboratory of Hemostasis, Division of Hematology Research and Review, Center for Biologics Evaluation and Research, Food and Drug Administration, Silver Spring, Maryland 20993, United States of America; 4 Center for Cancer and Blood Disorders, Children’s National Medical Center, Washington, D. C. 20010, United States of America; 5 School of Medicine and Health Sciences, George Washington University, Washington, D. C. 20037, United States of America; University of Lisbon, PORTUGAL

## Abstract

Hemophilia A (HA) is a bleeding disorder caused by deficiency of functional plasma clotting factor VIII (FVIII). Genetic mutations in the gene encoding FVIII (*F8*) have been extensively studied. Over a thousand different mutations have been reported in the *F8* gene. These span a diverse range of mutation types, namely, missense, splice-site, deletions of single and multiple exons, inversions, etc. There is nonetheless evidence that other molecular mechanisms, in addition to mutations in the gene encoding the FVIII protein, may be involved in the pathobiology of HA. In this study, global small ncRNA expression profiling analysis of whole blood from HA patients, and controls, was performed using high-throughput ncRNA microarrays. Patients were further sub-divided into those that developed neutralizing-anti-FVIII antibodies (inhibitors) and those that did not. Selected differentially expressed ncRNAs were validated by quantitative reverse transcription-polymerase chain reaction (qRT-PCR) analysis. We identified several ncRNAs, and among them hsa-miR-1246 was significantly up-regulated in HA patients. In addition, miR-1246 showed a six-fold higher expression in HA patients without inhibitors. We have identified an miR-1246 target site in the noncoding region of F8 mRNA and were able to confirm the suppressory role of hsa-miR-1246 on *F8* expression in a stable lymphoblastoid cell line expressing FVIII. These findings suggest several testable hypotheses vis-à-vis the role of nc-RNAs in the regulation of *F8* expression. These hypotheses have not been exhaustively tested in this study as they require carefully curated clinical samples.

## Introduction

Hemophilia A (HA) is an X chromosome-linked bleeding disorder, caused by mutations in the *F8* gene that results in a dysfunctional clotting Factor VIII (FVIII) [1]. HA can be effectively managed with regular infusions of plasma-derived- or recombinant-FVIII [2] but the development of anti-drug antibodies that inhibit FVIII function (inhibitors), which occurs in 20–30% of patients, is a significant impediment to successful treatment [3]. Despite over two decades of progress and improvements in the FVIII drug-products including the introduction of recombinant and bioengineered products, there has been no decrease in the prevalence of inhibitors among HA patients. It has also become increasingly clear that the underlying genetics of individual patients represent risk-factors for the development of inhibitors [4]. The diversity of HA mutations and their association with both disease severity and the development of inhibitors is captured, e.g. in a database compiled by the CDC (CDC Hemophilia A Mutation Project or CHAMP). These studies, and the resultant databases [5], have to-date focused largely on genetic defects in the *F8* gene and, more recently, on the HLA repertoire of individual patients [6].

Studies on *F8* gene defects and disease severity or inhibitor status often include a small but significant subset of patients in which no mutations can be detected in the coding sequence. These patients are excluded in the analysis and the cause for the disease is not discussed. As a case in point, we consider the CHAMP database (http://www.cdc.gov/ncbddd/hemophilia/champs.html) with 677 genotyped HA patients. No mutations were detected in 23 patients and an additional 5 had only synonymous mutations. Thus, over 4% of patients had no changes in the primary amino acid sequence of FVIII and still manifested the disease (Table 1). Other mutation types that occur at comparable frequencies such as deletions and mutations that affect splice-sites have been extensively studied. It is particularly intriguing that patients with no mutations exhibit the complete range of HA phenotypes, from mild to severe with over 50% of the patients being severe. Similarly, individual patients with the same mutation (or class of mutations) exhibit differing severities of the disease. Observations such as these, suggest that mechanisms other than mutations in the coding sequence *per se* may play either a primary or secondary role in the manifestation of HA.

**Table 1:** Occurrence of HA mutation types and prevalence of inhibitors in each class.

HA Mutation type	INH-*	INH+*	Total	% INH+*	*f* #
**Frame-shift**	**63**	**8**	**71**	**11.27**	**10.61**
**Deletion**	**14**	**12**	**26**	**46.15**	**3.89**
**Inversion**	**131**	**49**	**180**	**27.22**	**26.91**
**Missense**	**267**	**17**	**284**	**5.99**	**42.45**
**Nonsense**	**47**	**13**	**60**	**21.67**	**8.97**
**Splice-site**	**15**	**5**	**20**	**25.00**	**2.99**
**NONE**	**20**	**3**	**23**	**13.04**	**3.44**
**SYNONYMOUS**	**5**	**0**	**5**	**0.00**	**0.75**

* INH- and INH+, Number inhibitor negative and positive respectively

^#^ Frequency (*f*) in the HA cohort expressed as a percent (%). Source: CHAMP database

The expression of proteins of the coagulation pathway needs to be well regulated because either up-regulation or down-regulation can result in adverse clinical outcomes. While low levels of or non-functional FVIII causes HA, high levels of FVIII have been well established to be a risk-factor for thrombosis [7, 8]. In addition to *F8* gene, other molecular mechanisms have, to a limited extent, been explored. In a small cohort study of patients with acquired HA, differentially expressed genes between inhibitor and non-inhibitor patients were identified using gene expression microarray analysis [9]. Moreover, while very low level of FVIII in plasma is indicative of severe HA, another recent study indicates that platelets in circulation are in a pre-activated state in severe HA patients and these patients were observed to consume lower amounts of FVIII in therapy [10]. This finding suggests that, to some extent, the activated platelets in this group of patients compensate for the very low levels of FVIII. This further supports the idea that while mutations in *F8* are the basis for HA disease, severity or phenotypic expression of the disease may also be regulated by other molecular mechanisms, such as by suppression of F8 expression

In the past decades, non-coding RNAs (ncRNAs), i.e. RNA transcripts not translated into proteins, have emerged as key players in the regulation of gene expression. Examples of ncRNAs include transfer RNA (tRNA), ribosomal RNA (rRNA), microRNA (miRNA), small nucleolar RNA (snoRNA), small nuclear RNA (snRNA), piwi-interacting RNA (piRNA), small Cajal body-specific RNA (scaRNA), and long noncoding RNA (lncRNA). It has become increasingly clear that small ncRNAs, such as miRNAs and snoRNAs, are involved in a number of biological processes and the impairment of ncRNAs has been associated with many human diseases. MicroRNAs are endogenous single-stranded ncRNAs, 19–25 nucleotides in length that mediate transcriptional and post-transcriptional regulation of gene expression through mRNA degradation, inhibition of translational initiation, or inhibition of transcription [11–13] snoRNAs are usually 70–200 nucleotides in length and primarily mediate post-transcriptional modifications of other RNAs, especially rRNA, tRNA or snRNAs, through methylation or pseudouridylation (see reviews in [14, 15]).

This is the first survey of ncRNA expression in normal controls and HA patients with and without inhibitors. We demonstrate that several ncRNAs are differentially dysregulated in HA patients. One miRNA, the hsa-miR-1246, which was most significantly dysregulated, was characterized in additional detail. We have confirmed higher levels of miRNA by quantitative RT-PCR and demonstrate that hsa-miR-1246 regulates *F8* gene expression.

## Results

### Significantly dysregulated ncRNAs differentiate HA samples from controls

To identify significantly differentially expressed ncRNAs in HA patients, we isolated total RNA from whole blood from HA patients and healthy individuals, then conducted small ncRNA expression profiling analysis using a high-throughput microarray (see Methods). Prior to microarray analysis, the purity and quality of RNA samples were assessed to ensure the success and the reliability of microarray results. All RNA samples were found to have a 260/280 ratio higher than 1.8, indicating that the purity of RNA samples were suitable for microarray analysis (S2 File). In addition, a gel-on-chip analysis was performed to assess RNA integrity and the enrichment of low molecular weight RNAs. All RNA samples were separated into distinct bands corresponding to 28S and 18S ribosomal RNAs, as well as low molecular weight RNAs (Fig A in S1 File), indicating optimal integrity and high abundance of low molecular weight RNA species and thus deemed suitable for subsequent small ncRNA microarray analysis.

**Fig 1:**
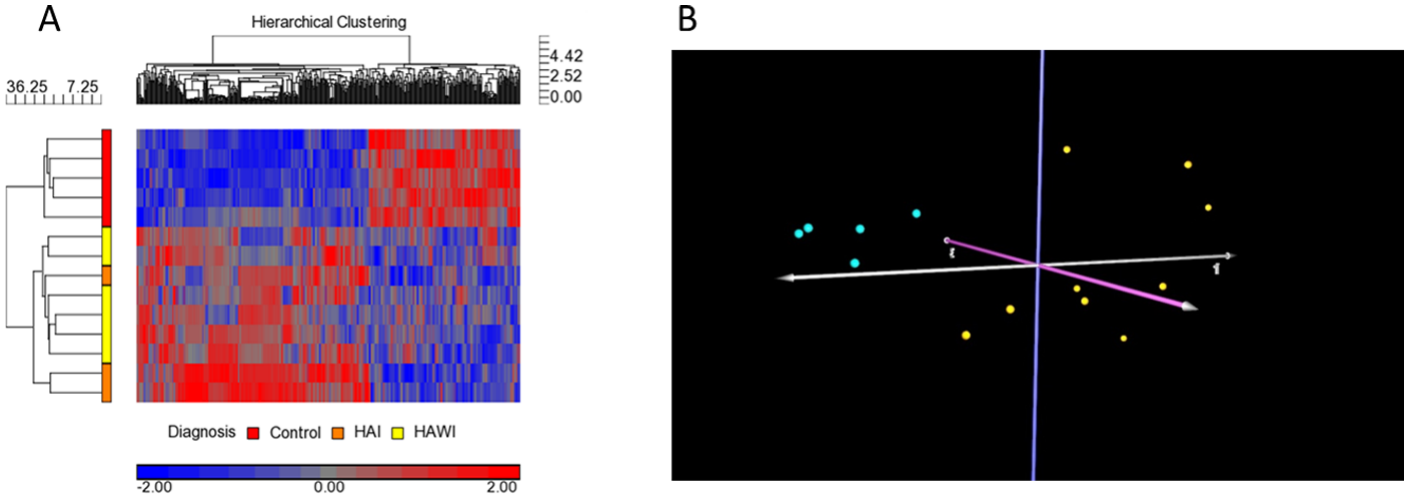
Hierarchical clustering and principal component analyses of significantly differentially expressed ncRNAs from ANOVA analysis. (A) Unsupervised hierarchical clustering analysis of 100 significantly differentially expressed ncRNAs between all hemophilia A patients (yellow and orange bars) and controls (red bar) shows the distinct ncRNA expression pattern of the two group (p-value < 0.01). Each row represents a subject, whereas each column is a ncRNA probe. Blue color in the heat map indicates down-regulation, whereas red indicates up-regulation. (B) Principal component analysis of samples on the basis of the 100 significantly differentially expressed ncRNAs identified by the ANOVA analysis described above. Yellow spheres represent HA samples, whereas turquoise spheres represent unaffected controls.

The RNA samples were poly(A)-tailed and labeled with biotin prior to hybridizing on ncRNA microarrays. An enzyme linked oligosorbent assay (ELOSA) was used to evaluate the success of the labeling processes (S3 File). The Expression Console software (Affymetrix) was employed to assess the success of the RNA labeling protocol and microarray processing after hybridization. The log2 signal intensity values of all spike-in control probes were higher than 9.96 in all RNA samples, confirming that the labeling and array processing were successful (Fig B in S1 File). The ncRNA microarray data were uploaded into the Partek Genomics Suite software for background subtraction, normalization, and statistical analysis. Successful normalization was determined by similar patterns of distribution box-and-whisker plots (Fig C in S1 File) and histograms (Fig D in S1 File) of signal intensity values across all samples. Significantly differentially expressed ncRNAs in the samples obtained from HA patients and healthy individuals were identified by ANOVA analysis of successfully normalized ncRNA microarray data. A 2-class ANOVA analysis revealed 100 human ncRNA probes that were significantly differentially regulated (p-value < 0.01) between all HA patients and healthy participants. These ncRNA probes and their corresponding fold-changes are shown in S4 File. Cluster analysis was performed to further determine whether or not the expression levels of these ncRNAs could distinguish between the HA and control groups. The hierarchical clustering analysis revealed a distinct separation of the HA patient and the control groups based on expression profiles of the differentially expressed ncRNAs (Fig 1A). Principal component analysis (Fig 1B), which was employed to reduce the dimensionality of the microarray data, also revealed clear separation between HA subjects and controls based on the 100 human ncRNA probes identified by the 2-class ANOVA.

### Significantly differentially expressed ncRNAs differentiate HA patients with and without inhibitors

**Fig 2:**
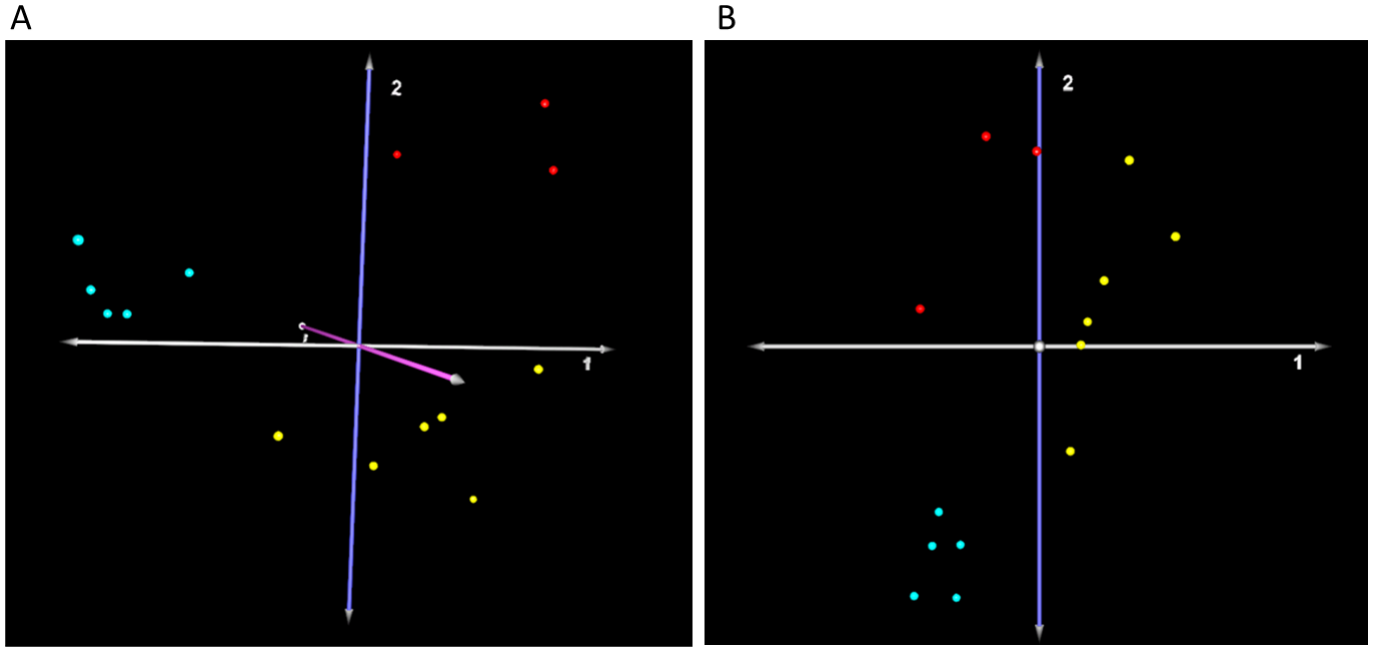
Principal component analysis of significantly differentially expressed ncRNAs from 3-class ANOVA analysis and 3-class SAM analysis. **(A)** 3-class ANOVA analysis. Principal component analysis of samples based on 88 significantly differentially expressed ncRNAs from 3-class ANOVA analysis (p-value < 0.01) reduces the dimentionality of the data and shows the clear separation between the hemophilia A patients with inhibitor development (red), the hemophilia A patients without inhibitor development (yellow), and the controls (turquoise). **(B)** 3-class SAM analysis. Principal component analysis of samples based on 11 significantly differentially expressed ncRNA probes from 3-class SAM analysis (FDR < 5%) reveals the distinct separation between the hemophilia A patients with inhibitor development (red), the hemophilia A patients without inhibitor development (yellow), and the controls (turquoise).

The most significant complication of treatment in patients with HA is the development of inhibitors and it is still unknown whether ncRNAs are involved in this condition. To identify significantly differentially expressed ncRNAs that differentiate between HA patients who develop inhibitory antibodies and those who do not, we further divided the samples from the HA subjects into two subgroups based on the presence or absence of inhibitors. A 3-class ANOVA analysis revealed as many as 88 human ncRNA probes that were significantly differentially regulated (p-value < 0.01) among hemophilia subgroups and controls. These ncRNA probes and their corresponding fold-changes are shown in S5 File. Principal component analysis (Fig 2A) also showed distinct separation between samples from HA patients with- and without inhibitors, based on the 88 significant probes.

**Fig 3:**
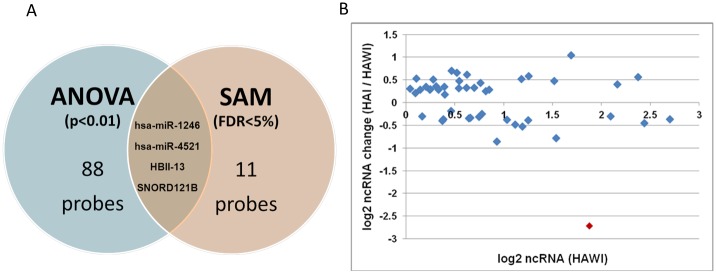
Differentially expressed ncRNA probes identified by 3-class ANOVA and SAM analyses. **(A)** Significant ncRNA probes were identified using 3-class ANOVA analysis with p-value < 0.01 and 3-class SAM analysis with FDR < 5%. A total of 4 ncRNAs (hsa-miR-1246, hsa-miR-4521, HBII-13, and SNORD121B) were found to overlap between the two statistical analyses. **(B)** Hsa-miR-1246 (red dot) is significantly down-regulated in HA patients with inhibitor relative to patients without inhibitor.

To increase the stringency of statistical significance analysis, the normalized ncRNA microarray data from Partek Genomics Suite software were also uploaded into the TMeV software and SAM analysis was conducted. A 3-class SAM analysis of the data revealed a total of 11 significantly differentially expressed ncRNAs probes with the false discovery rate less than 5% (Table 2). Consistent with their corresponding mature ncRNA products, several precursor ncRNAs (i.e. hp-hsa-miR-181d, hp-hsa-miR-1246, and hp-hsa-miR-4521) were also significantly differentially regulated. Principal component analysis of the subjects based on this set of significant probes also showed distinct separation between samples from HA patients and the controls, as well as between samples from HA patients with and without inhibitors (Fig 2B). Out of 11 ncRNAs identified by the 3-class SAM analysis (FDR < 5%), a total of 4 differentially expressed ncRNAs overlapped with the 3-class ANOVA (p-value < 0.01). These ncRNAs are hsa-miR-1246, hsa-miR-4521, HBII-13, and SNORD121B (Fig 3A).

**Table 2:** Significantly differentially regulated ncRNAs identified by SAM analysis. A 3-class SAM analysis of ncRNA microarray data revealed 11 ncRNA probes that were differentially expressed between hemophilia A (HA) patients with inhibitor development (HAI), hemophilia A patients without inhibitor development (HAWI), and controls (C) with the false discovery rate less than 5%.

Transcript ID	Fold-Change (all HA vs. C)	Fold-Change (HAI vs. C)	Fold-Change (HAWI vs. C)	Fold-Change (HAI vs. HAWI)
hsa-mir-1246	5.001	1.949	12.834	-6.585
HBII-13	2.538	3.646	1.766	2.065
hsa-mir-4521	2.288	2.885	1.814	1.591
HBII-13	2.200	3.193	1.516	2.106
hsa-mir-181d	1.951	2.234	1.703	1.312
SNORD121B	1.498	1.701	1.318	1.290
SNORD121B	1.429	1.551	1.317	1.177
hp-hsa-mir-181d	1.229	1.411	1.071	1.317
hp-hsa-mir-1246	1.181	1.198	1.163	1.030
hp-hsa-mir-181d	1.113	1.201	1.032	1.164
hp-hsa-mir-4521	1.067	1.095	1.039	1.054

### TaqMan Quantitative RT-PCR confirmation of differentially regulated ncRNAs

The ncRNAs (i.e. hsa-miR-1246, hsa-miR-4521, HBII-13, and SNORD121B) that were significantly differentially expressed (p-value < 0.01; FDR < 5%) were selected for validation by ncRNA TaqMan quantitative RT-PCR (qRT-PCR) analyses. The expression levels of hsa-miR-1246, hsa-miR-4521, and HBII-13 from qRT-PCR analyses were correlated with ncRNA microarray data, confirming the up-regulation of these ncRNAs in samples from HA patients (Table 3). The amplification of SNORD121B was not successful by the TaqMan qRT-PCR assays that we used. Moreover, the results demonstrate that while hsa-mir-1246 is up-regulated in samples from HA patients compared to healthy donors, the average level of hsa-miR-1246 in HA patients without inhibitors is over 10-fold higher than those patients with inhibitors.

**Table 3:** Results of TaqMan ncRNA qRT-PCR analysis of differentially regulated ncRNAs. All cases (n = 9) and controls (n = 5) that were used for ncRNA microarray analysis were included in TaqMan ncRNA qRT-PCR analysis.

ncRNA	Fold-Change(all HA vs. C)		Fold-Change(HAI vs. C)		Fold-Change(HAWI vs. C)	
	Microarray	qRT-PCR (p-value)	Microarray	qRT-PCR (p-value)	Microarray	qRT-PCR (p-value)
hsa-miR-1246	5.001	30.056 (0.382)	1.949	3.107 (0.146)	12.834	43.534 (0.297)
hsa-miR-4521	2.288	2.328 (0.014)	2.885	2.173 (0.013)	1.814	2.656 (0.027)
HBII-13	2.538	3.357 (0.006)	3.646	3.127 (0.047)	1.766	3.110 (0.066)

**Fig 4:**
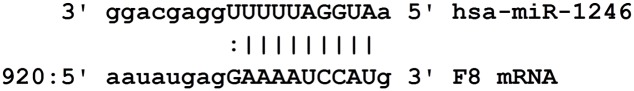
Sequence alignment of hsa-miR-1246 and *F8* mRNA.

The patient sample sizes in the small RNA expression profiling are small but comparable to those used in such profiling studies (). However to vailidate the up-regulation of hsa-miR-1246 in HA patients we performed PCR based assays on a total of 15 HA patients. We have included the results of both *F8* mRNA levels and expression of has-miR-1246 (Fig 4).

### Regulation of *F8* gene expression by hsa-mir-1246

**Fig 5:**
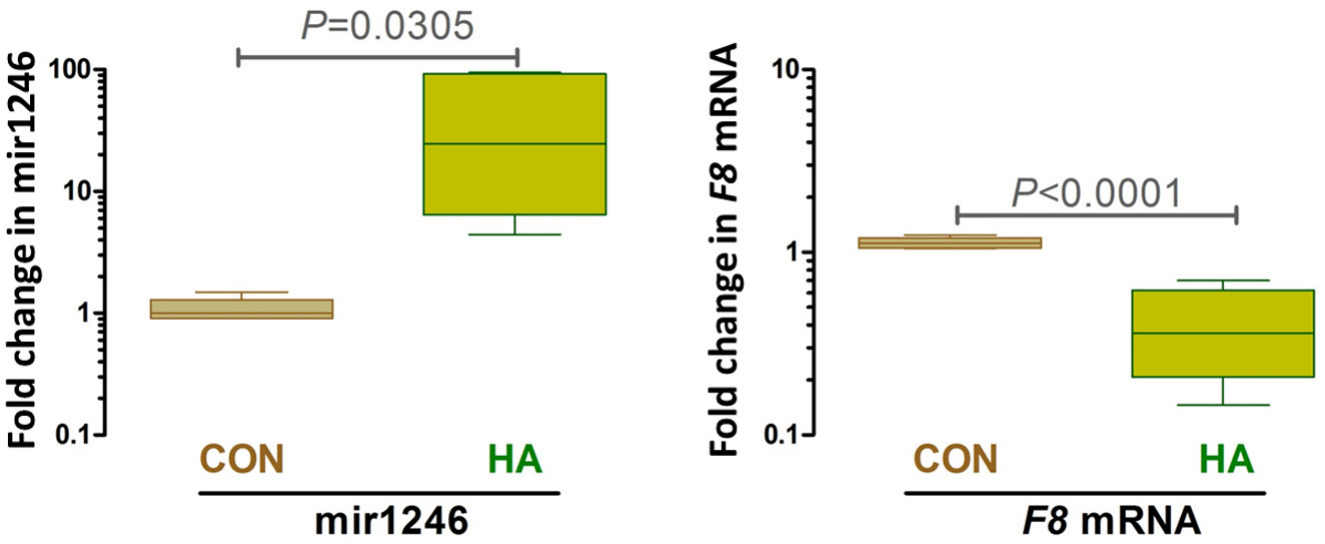
Fold change in hsa-miR 1246 and F8 mRNA levels in HA patients compared to normal donors. (**A**) The fold change in has-miR 1246 was determined by RT-PCR. Total RNA was isolated from the blood of 5 control (CON) donors and 15 HA patients (HA). There is a significant increase in the has-miR 1246 levels in samples from HA patients compared to controls. (**B**) The fold change in *F8* mRNA was determined by RT-PCR. Total RNA was isolated from the blood of 5 control (CON) donors and 15 HA patients (HA). There is a significant decrease in the *F8* mRNA levles in samples from HA patients compared to controls.

To determine whether the significantly up-regulated hsa-miR-1246 can bind *F8* mRNA transcript and regulate *F8* expression post-transcriptionally, we have scanned *F8* gene mRNA for the miR-1246 binding site using TargetScan Human Database, Release 6.0 (http://www.targetscan.org). Interestingly, we found that the base positions 931–937 of *F8* 3’UTR (5’-…GAGGAAAAUCCAUG…-3’) exactly matched the reverse complementary sequence of the base positions 2–8 (i.e. the seed region) of the mature hsa-miR-1246 sequence (5’-AAUGGAUUUUUGGAGCAGG-3’), clearly suggesting that *F8* mRNA is a putative target of hsa-miR-1246 (Fig 5).

To further determine that in fact hsa-miR-1246 regulates *F8*, we performed a functional analysis by transfecting hsa-miR-1246 precursor into FVIII-expressing cells. We have previously characterized lymphoblastoid cells derived from a healthy donor and an HA patient extensively. The cell line derived from the healthy donor (*Nor*) was shown to express both *F8* mRNA transcript and FVIII protein [6]. We also showed that the cell lines can be used in transfection experiments, specifically siRNA-mediated knockdown of endogenous FVIII. Here, we overexpressed hsa-miR-1246 expression in the *Nor* lymphoblastoid cell line by transfecting the cells with hsa-miR-1246 miRNA Precursor or Negative Control miRNA Precursor at a manufacturer recommended concentration of 10 mM. TaqMan qRT-PCR assays revealed a significant up-regulation of hsa-miR-1246 in the transfected cells with hsa-miR-1246 miRNA Precursor relative to Negative Control miRNA Precursor (Table 4), demonstrating the successful overexpression of hsa-miR-1246 mediated by transfection. Interestingly, the level of *F8* transcript in the cells overexpressed hsa-miR-1246 was significantly reduced compared with the cells transfected with Negative Control miRNA Precursor (Table 4), indicating that hsa-miR-1246 regulates *F8* mRNA expression.

**Table 4:** Regulation of *F8* mRNA levels in *Nor* lymphoblastoid cells. Fold change in levels of mir-1246 and *F8* mRNA were determined in *Nor* lymphoblastoid cells using a functional assay. The cells were transfected with the hsa-miR-1246 miRNA Precursor or Negative Control miRNA Precursor at a manufacturer recommended concentration of 10 mM. Fold change in the miR-1246 and *F8* mRNA was monitored.

Mir-1246 precursor	Fold-Change in miR-1246 (with respect to non-targeted miR)		Fold-Change in *F8* mRNA (with respect to non-targeted miR)	
0 nM	1		1.00	
10 nM	1.5 ± 0.05	P = 0.0005	0.74 ± 0.02	P < 0.0001

## Discussion

In this study, we have investigated whether differences in ncRNA expression profiles could be discerned between HA patients and normal subjects, as well as between patients who did, and did not develop inhibitors to FVIII. We identified 100 significant ncRNAs (S4 File) differentially expressed in HA patients compared to normal controls (p < 0.01). Unsupervised hierarchical clustering and principal component analyses of these ncRNAs (Fig 2) demonstrate a clear separation between HA patients and normal controls, as well as between the HA subgroups. The results of an unbiased approach, such as the one outlined above, is generally more successful than a candidate gene association study when the genetic basis of a disease is unknown [16]. In the case of HA, however, a large body of literature has clearly established that this genetic disease is caused by decreased FVIII activity [17]. Nonetheless, given the large diversity of individual mutations (well over a thousand) in the *F8* gene (http://hadb.org.uk/), it is not clear that every HA case is caused by a functionally defective FVIII protein *per se*. Many HA patients are identified as CRM negative, i.e. no FVIII antigen is detected in the plasma. For some mutations, this can be attributed to the mis-folded FVIII not being secreted from cells [18]. However, as described in the introduction, genetic characterizations of HA patients often identify some patients in whom no mutation in the FVIII coding sequence can be detected (Table 1 and [19]). Finally individual patients with the same (or similar) genetic mutation present HA with different severities. It is thus plausible that negative regulators (e.g. ncRNAs), which have received almost no attention in research studies on HA, appear to have important functions associated with the clinical manifestation of this disease.

The diversity in the clinical severity of HA is likely to be driven by the variability in the genetic defects of *F8* gene, as well as those elements that control transcription. Thus, for example, in patients who are truly CRM negative (i.e. do not synthesize any FVIII), ncRNAs could play either a primary or a secondary role in the lower expression of FVIII. Based on our microarray data, hsa-miR-1246 was found to exhibit the largest fold-increase (12.8) in the expression of ncRNA in samples from HA patients without inhibitors compared to control samples (Table 2). This increased expression of hsa-miR-1246 was further confirmed by qRT-PCR analysis (Table 3 and Fig 4) and may be important in regulating *F8* expression. The *F8* gene is a potential target for hsa-miR-1246 (mirSVR score, -0.2829; PhasCons score, 0.5303 (www.microrna.org)). As these patients receive regular prophylactic infusions of FVIII measuring FVIII antigen levels in these patients would not be informative. We have estimated levels of *F8* mRNA and has-miR-1246 in 15 HA patients. The box and whisker plot shown in Fig 5 shows that the general trend is that patients with HA have significantly higher (P = 0.0305) levels of has-miR-1246 and lower (P<0.0001) levels of *F8* mRNA compared to control donors without HA. We would caution however that the *F8* mutations *per se* will result in lower mRNA levels in many patients. Thus, there is no direct correlation between *F8* mRNA and has-miR-1246 levels in individual patients. It is however conceivable that up-regulation of hsa-miR-1246 is associated with lower levels of FVIII. The overall spread of the data with respect of levels of hsa-miR-1246 suggests that the relative effect of this nc-RNA in disese manifestation differs in individual patients. We will need very well characterized clinical samples with specific genetic characteristics to elucidate the precise role of hsa-miR-1246 in HA pathobiology. We will conduct such studies in the future and hope others with greater access to clinical samples will do so too.

We also observed a 6.585-fold decrease in hsa-miR-1246 expression in HA patients with inhibitors when compared to patients without inhibitors (Table 2). This is intriguing because when one considers HA patients without inhibitors, there is a 12.834-fold increase in the expression of hsa-miR-1246 (see above and Table 2).

It is a common rule that an ncRNA will target more than one gene. The profiling of ncRNAs from natural killer cells has shown that hsa-miR-1246 is down-regulated and associated with the stimulation of IL-2, IL-15, and IL-21. These cytokines however are also the key players in T-cell responses and the lower levels of hsa-miR-1246 in blood samples of HA patients who have had a history of anti-drug antibodies is very significant. A large body of literature has accumulated in recent years to demonstrate an association between HA-causing *F8*-mutation-types and individual responses vis-à-vis immunogenicity (for a meta-analysis see [4]). These data are consistent with the percept that synthesis of the FVIII polypeptide chain is necessary for inducing central tolerance. For example, HA patients who have a missense mutations in the *F8* gene have a <10% life-time prevalence of inhibitors, whereas prevalence of inhibitors in individuals with large gene deletions can be as high as 88%. The *F8* genotype can thus explain many but not all the differences in the individual immunological responses to infused FVIII. The results of this study thus argue for an additional mechanism, mediated by ncRNAs, in regulating the development of antibodies in response to FVIII infusions, which warrants further study.

Our data show a distinct 3-way separation of ncRNAs expressed in normal donors and HA patients who do and do not develop inhibitors. In addition, we have identified hsa-miR-1246 as potentially having important roles in regulating both FVIII levels and the immune response to FVIII-infusions in HA patients. The mechanistic basis for the former was investigated using the *Nor* cell-line, in which we have previously demonstrated expression of *F8* mRNA and FVIII protein [6]. Transfection of these cells with hsa-miR-1246 miRNA precursor showed elevated levels of hsa-miR-1246 and reduced levels of *F8* mRNA by qRT-PCR. These data show that hsa-miR-1246 can indeed regulate FVIII levels.

This study strongly suggests that ncRNAs play a significant role in regulating FVIII levels, the dysregulation of which can have adverse clinical consequences. The main site of FVIII expression is the liver. We were unable to estimate miR-1246 levles in the liver because we were unable to obtain human liver biopsies that are suitable for extraction of mRNA for our needs. However, miR-1246 is known to be expressed in human liver cell lines such as HepG2, Hep 11 and Hep 12 (Hibino et al, Oncogenesis. 2014; Sun et al, BMC Cancer. 2014). In addition, ncRNAs may contribute to variable responses to FVIII-replacement therapy, particularly with respect to the development of inhibitors. These specific clinical outcomes will of course have to be confirmed in additional studies on carefully selected patient samples. This will be challenging because such patients will in many instances constitute a rare sub-population for a rare disease. Another intriguing hypothesis generated by this study is that ncRNAs may be potential biomarkers for deep-vein thrombosis. Although numerous studies have linked high FVIII levels to deep-vein thrombosis [8, 20, 21] and subjects with FVIII concentrations above 1500 IU/L have an adjusted odds ratio of 4.8, a mechanistic explanation is lacking. Our finding that hsa-miR-1246 can regulate *F8* expression thus provides a testable hypothesis; that hsa-miR-1246 is down regulated in individuals with high FVIII serum levels. Testing this hypothesis however remains outside the scope of this study.

This first systematic survey of ncRNA profiles in HA patients indicates that besides the coding sequence of *F8*, non-coding regions and other regulatory elements should also be studied to fully understand the causes and consequences of dysregulation of *F8* gene expression. We however emphasize that this study serves principally to generate novel testable hypotheses.

## Materials and Methods

### Sample Collection

In compliance with local Institutional Review Board regulations, 15 patients with HA were enrolled in this study. All of them were being managed by the Hemophilia Treatment Center at Children’s National Medical Center, Washington DC. Patients included in the study were 9 months– 30 years of age and had a diagnosis of HA confirmed by FVIII levels. Patients with and without inhibitors to FVIII were included in the study. After informed consent and approval, 1–1.5 mL venous blood was collected in buffered sodium citrate tubes from each participant. Soon after collection, RNAlater Solution (Life Technologies, Grand Island, NY, USA) was added to each blood sample and samples stored at -20°C until ready for RNA isolation.

Clinical data was collected by patient/parent interview and review of medical charts. For the control group, whole blood samples were collected from healthy individuals (n = 5) from the NIH Blood Bank (Bethesda, MD, USA).

### RNA isolation

Total RNA was isolated and purified from whole blood using the RiboPure Blood Kit (Life Technologies) according to the manufacturer’s protocol with modifications to enhance the enrichment of low molecular weight RNAs. Briefly, whole blood in the RNAlater Solution was centrifuged and the pellet mixed with 800 μl Lysis Solution (Life Technologies). A total of 10 μl acetic acid was added to the lysates and total RNA was extracted by adding 500 μl acid-phenol chloroform. Following centrifugation, the aqueous (upper) phase was carefully transferred to a 15 ml RNase-free tube and total RNA was then precipitated by adding 2.7 ml of 100% ethanol. RNA was purified using a RNA Filter Cartridge (Life Technologies). Purified RNA on the filter was then eluted by adding 100 μl Elution Solution (Life Technologies) containing 0.1 mM EDTA. RNA concentration was measured using the NanoDrop 1000 spectrophotometer (Thermo Scientific, Waltham, MA, USA) and the purity of RNA was determined by the 260/280 ratio of higher than 1.8. All RNA samples were analyzed by a gel-on-chip analysis using Agilent Bioanalyzer to assess RNA integrity and the enrichment of low molecular weight RNAs.

### Small non-coding RNA microarray analysis

All RNA samples were poly (A)-tailed and biotin-labeled using the FlashTag Biotin HSR RNA Labeling Kit (Affymetrix, Santa Clara, CA, USA). After labeling, the enzyme linked oligosorbent assays (ELOSA) were performed to confirm that the biotin labeling processes were successful. Biotin-labeled RNA samples (500 ng/sample) were hybridized on GeneChip miRNA 3.0 microarrays (Affymetrix), each of which contains 19,724 probe sets covering over 5,600 human miRNAs, pre-miRNAs, snoRNAs, and scaRNAs, whose sequence information is based on the miRBase database version 17 http://www.mirbase.org/. After hybridization, the hybridization cocktail was extracted from each microarray and all microarrays were automatically washed and stained using the Fluidics Station 450 (Affymetrix). The microarrays were scanned using GeneChip Scanner 3000 7G (Affymetrix). All washing, staining, and scanning steps were controlled by the Affymetrix Command Console software according to the manufacturer’s instruction for GeneChip miRNA 3.0 microarrays. The Expression Console software (Affymetrix) was then used to evaluate the success of the labeling protocol and array processing. Partek Genomics Suite software was used to analyze the intensities for the probe elements across the microarray and to identify significantly differentially expressed ncRNAs. The data normalization procedures for Affymetrix miRNA microarrays include RNA background correction, quantile normalization, log (base 2) transformation of intensity data, and probe set summarization using Median Polish method. Analysis of variance was then used for identifying significantly differentially expressed ncRNAs in HA according to the Partek Genomics Suite workflow recommended by Affymetrix.

The normalized data from Partek program were also uploaded into the TIGR Multiexperiment Viewer (TMeV) software package to perform other statistical analyses on the microarray data [22, 23]. The Significance Analysis of Microarrays (SAM) was conducted and the percentage of the false discovery rate for each ncRNA was calculated [24, 25].

### TaqMan qRT-PCR analysis

To validate the microarray data, ncRNAs that were significantly differentially expressed in HA patients were selected for confirmation analysis by small ncRNA TaqMan quantitative reverse transcription polymerase chain reaction (qRT-PCR) analysis using the TaqMan MicroRNA Assays or the Custom TaqMan Small RNA Assays (Life Technologies). According to the manufacturer’s TaqMan Assay protocol, cDNA was reverse transcribed from 10 ng of total RNA using specific looped small RNA RT primers which allow for specific RT reactions that can discriminate mature small ncRNA sequences from their precursors. The cDNA was then amplified by PCR, which uses TaqMan minor groove binder probes containing a reporter dye (FAM dye) linked to the 5' end of the probe, a minor groove binder at the 3' end of the probe, and a non-fluorescence quencher at the 3' end of the probe. The design of these probes allows for more accurate measurement of reporter dye contributions than possible with conventional fluorescence quenchers. Relative expression of ncRNAs was determined using delta-delta Ct (ΔΔCt) method. Hsa-miR-106b was used as an endogenous control in all qRT-PCR experiments because our microarray analysis shows that it is abundantly expressed in the blood, has very little variance across samples, and is not significantly differentially expressed in RBCs from HA patients.

### Statistical analysis

In this study, a 3-class ANOVA analysis was conducted to determine whether or not the means of ncRNA expression in HAI, HAWI, and control groups are statistically significantly different by calculating P-values. The null hypothesis in this study is that the average expression levels of a given ncRNA in all groups (i.e. HAI, HAWI, and control groups) are the same. If a probability (p-value) is less than a significance level (p-value < 0.05), we reject the null hypothesis, meaning the average expression level of a given ncRNA in these groups is significantly different.

A 3-class SAM analysis was conducted with a similar purpose which is to determine whether or not the expression of each ncRNA is statistically significantly different in HAI, HAWI, and control groups. However, the algorithm used for identifying significant ncRNAs is different. SAM analysis employs non-parametric statistics as the data may not show a normal distribution resulting in a significant ncRNA list that is different from ANOVA and also allow us to set the level of false discovery rate. For details about SAM test see [Chu, G., B. Narasimhan, R. Tibshirani and V. Tusher (2002). SAM "Significance Analysis of Microarrays" Users Guide and Technical Document. http://www-stat.stanford.edu/~tibs/SAM/]

Generally only one or the other of these statistical tools (i.e. ANOVA or SAM) are employed in microarray analyses. We have performed both analyses separately and then identified the overlap between the two analysis to increase the stringency which allows for identification of candidate ncRNAs with high fidelity.

### Microarry data reporting

The microarray dataset has been submitted to the GEO repository and has been assigned the accession number GSE65581.

### Ethics Statement

The study protocol was approved by the Institutional Review Board (#Pro00003130) at Children’s National Medical center in Washington, DC where the samples were collected and was also approved by the Food and Drug Administration’s Research Involving Human Subjects Committee (RIHSC# 12-118B) where the samples were processed and analyzed for the study described here. All subjects who involved in the study gave written informed consent, which conformed to the current Helsinki Declaration.

## Supporting Information

S1 File Quality control assessment of RNA labeling and microarray processing. A gel-on-chip analysis of total RNA isolated from whole blood from individuals with hemophilia A with inhibitor (HAI) and without inhibitor (HAWI), as well as controls (C) was conducted to assess RNA integrity and the enrichment of small RNAs (arrow) in the samples (**Fig A**). To evaluate the success of the RNA labeling protocol and microarray processing, the spike-in labeling control probe sets were analyzed using the Expression Console software after data summarization and normalization. The spike-in control-2, -23, and -29 are RNA and confirm the poly (A) tailing and ligation. The spike-in control 31 is poly (A) RNA and confirms ligation. The spike-in control 36 is poly (A) DNA and confirms ligation and the lack of RNases in the RNA sample. Successful RNA labeling and microarray processing were determined by log2 signal greater than or equal to 9.96. The log2 signal intensity values of all spike-in probes are higher than 13.33 in all RNA samples (**Fig B**). The RNA samples were hybridized onto microarrays. After scanning and background subtraction, microarray data were normalized using quantile normalization method. Successful normalization was assessed by similar patterns of the distribution boxplots (**Fig C**) and histograms (**Fig D**) across all samples.
(TIF)

S2 File Spectrophotometry analysis of total RNA isolated from whole blood samples. Small RNA-enriched total RNA isolated and purified from whole blood stored in the RNAlater Solution was diluted in 100 μl of 0.1 mM EDTA. The samples were then analyzed using a Nanodrop spectrophotometer to measure the concentration and the purity of the RNA samples. The 260/280 ratio of 1.8 was used as a cutoff for the purity.
(DOCX)

S3 File Enzyme Linked Oligosorbent Assays (ELOSA) of biotin-labeled RNA samples. All small RNA-enriched total RNA samples were poly (A)-tailed and biotin-labeled. After labeling, the enzyme linked oligosorbent assays (ELOSA) were performed to confirm that the biotin labeling processes were successful. The absorbance at 450 nm of greater than 0.10 OD over a negative control was considered positive.
(DOCX)

S4 File Significantly differentially expressed ncRNAs between hemophilia A cases and controls identified by 2-class ANOVA (*P* value < 0.01). (DOCX)

S5 File Significantly differentially expressed ncRNAs between hemophilia A with- and without inhibitor development and controls identified by 3-class ANOVA (*P* value < 0.01). (DOCX)
