# Supplementary figures and images for: Small ncRNA Expression-Profiling of Blood from Hemophilia A Patients Identifies miR-1246 as a Potential Regulator of Factor 8 Gene
Source: PLoS One. 2015 Jul 15;10(7):e0132433. doi: 10.1371/journal.pone.0132433 (PMC4503767; doi:10.1371/journal.pone.0132433)

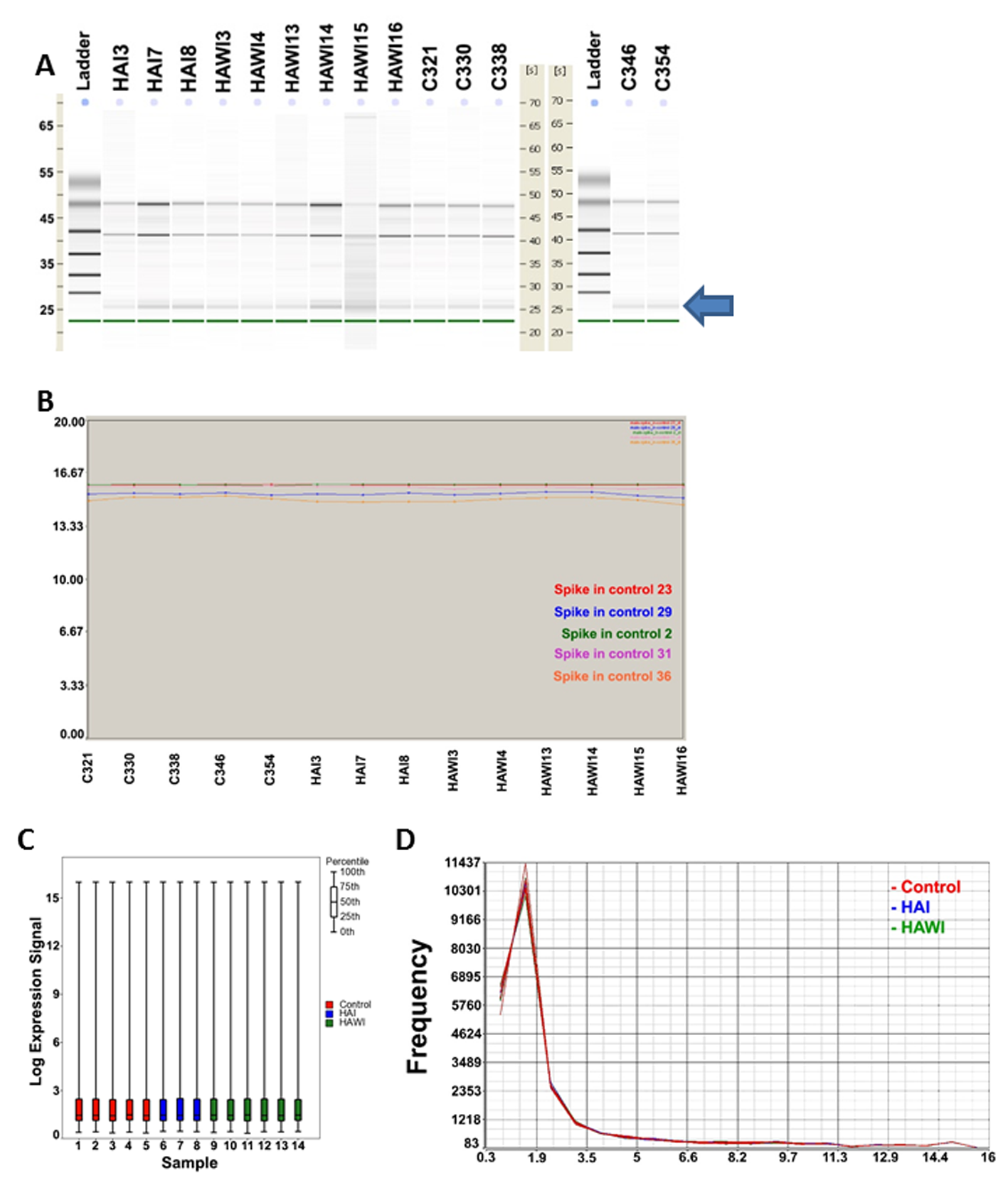

Supplement: S1 File — A gel-on-chip analysis of total RNA isolated from whole blood from individuals with hemophilia A with inhibitor (HAI) and without inhibitor (HAWI), as well as controls (C) was conducted to assess RNA integrity and the enrichment of small RNAs (arrow) in the samples (Fig A). To evaluate the success of the RNA labeling protocol and microarray processing, the spike-in labeling control probe sets were analyzed using the Expression Console software after data summarization and normalization. The spike-in control-2, -23, and -29 are RNA and confirm the poly (A) tailing and ligation. The spike-in control 31 is poly (A) RNA and confirms ligation. The spike-in control 36 is poly (A) DNA and confirms ligation and the lack of RNases in the RNA sample. Successful RNA labeling and microarray processing were determined by log2 signal greater than or equal to 9.96. The log2 signal intensity values of all spike-in probes are higher than 13.33 in all RNA samples (Fig B). The RNA samples were hybridized onto microarrays. After scanning and background subtraction, microarray data were normalized using quantile normalization method. Successful normalization was assessed by similar patterns of the distribution boxplots (Fig C) and histograms (Fig D) across all samples. (TIF) [file pone.0132433.s001.tif]
