# Supplementary material for: Small ncRNA Expression-Profiling of Blood from Hemophilia A Patients Identifies miR-1246 as a Potential Regulator of Factor 8 Gene
Source: PLoS One. 2015 Jul 15;10(7):e0132433. doi: 10.1371/journal.pone.0132433 (PMC4503767; doi:10.1371/journal.pone.0132433)
Supplement: S2 File — Small RNA-enriched total RNA isolated and purified from whole blood stored in the RNAlater Solution was diluted in 100 μl of 0.1 mM EDTA. The samples were then analyzed using a Nanodrop spectrophotometer to measure the concentration and the purity of the RNA samples. The 260/280 ratio of 1.8 was used as a cutoff for the purity. (DOCX) [file pone.0132433.s002.docx]

**Supplemental Table 1.** **Spectrophotometry analysis of total RNA isolated from whole blood samples.** Small RNA-enriched total RNA isolated and purified from whole blood stored in the RNAlater Solution was diluted in 100 µl of 0.1 mM EDTA. The samples were then analyzed using a Nanodrop spectrophotometer to measure the concentration and the purity of the RNA samples. The 260/280 ratio of 1.8 was used as a cutoff for the purity.

|  | | **Sample number** | **ng/µl** | **280/260** |
| --- | --- | --- | --- | --- |
| HA patients | HA with inhibitor | HAI 003 | 61.18 | 1.90 |
|  |  | HAI 007 | 113.93 | 1.95 |
|  |  | HAI 008 | 125.02 | 1.88 |
|  | HA without inhibitor | HAWI 003 | 68.16 | 1.89 |
|  |  | HAWI 004 | 66.79 | 1.89 |
|  |  | HAWI 0013 | 77.83 | 1.88 |
|  |  | HAWI 0014 | 83.98 | 1.95 |
|  |  | HAWI 0015 | 67.96 | 1.91 |
|  |  | HAWI 0016 | 115.70 | 1.89 |
| Normal donors | Control | C321 | 66.53 | 1.91 |
|  |  | C330 | 95.09 | 1.90 |
|  |  | C338 | 75.32 | 1.91 |
|  |  | C346 | 135.62 | 1.87 |
|  |  | C354 | 80.31 | 1.88 |
