# Supplementary material for: Small ncRNA Expression-Profiling of Blood from Hemophilia A Patients Identifies miR-1246 as a Potential Regulator of Factor 8 Gene
Source: PLoS One. 2015 Jul 15;10(7):e0132433. doi: 10.1371/journal.pone.0132433 (PMC4503767; doi:10.1371/journal.pone.0132433)
Supplement: S3 File — All small RNA-enriched total RNA samples were poly (A)-tailed and biotin-labeled. After labeling, the enzyme linked oligosorbent assays (ELOSA) were performed to confirm that the biotin labeling processes were successful. The absorbance at 450 nm of greater than 0.10 OD over a negative control was considered positive. (DOCX) [file pone.0132433.s003.docx]

**Supplemental Table 2. Enzyme Linked Oligosorbent Assays (ELOSA) of biotin-labeled RNA samples.** All small RNA-enriched total RNA samples were poly (A)-tailed and biotin-labeled. After labeling, the enzyme linked oligosorbent assays (ELOSA) were performed to confirm that the biotin labeling processes were successful. The absorbance at 450 nm of greater than 0.10 OD over a negative control was considered positive.

| **Sample ID** | **Absorbance** | **Result** |
| --- | --- | --- |
| HAI003 | 0.880 | Positive |
| HAI007 | 0.786 | Positive |
| HAI008 | 0.725 | Positive |
| HAWI003 | 0.605 | Positive |
| HAWI004 | 0.786 | Positive |
| HAWI013 | 0.733 | Positive |
| HAWI014 | 0.705 | Positive |
| HAWI015 | 0.727 | Positive |
| HAWI016 | 0.803 | Positive |
| C321 | 0.571 | Positive |
| C330 | 0.653 | Positive |
| C338 | 0.572 | Positive |
| C346 | 0.562 | Positive |
| C354 | 0.573 | Positive |
| Pos Control | 1.889 | Positive |
| Neg Control | 0.158 | Negative |
