# Supplementary material for: Small ncRNA Expression-Profiling of Blood from Hemophilia A Patients Identifies miR-1246 as a Potential Regulator of Factor 8 Gene
Source: PLoS One. 2015 Jul 15;10(7):e0132433. doi: 10.1371/journal.pone.0132433 (PMC4503767; doi:10.1371/journal.pone.0132433)
Supplement: S4 File — (DOCX) [file pone.0132433.s004.docx]

**Supplemental Table 3.** Significantly differentially expressed ncRNAs between hemophilia A cases and controls identified by 2-class ANOVA (*P* value < 0.01).

| **Transcript ID** | ***P* value** | **Fold-Change** |
| --- | --- | --- |
| hsa-mir-597 | 9.13E-05 | -1.180 |
| hsa-mir-107 | 1.25E-04 | 1.130 |
| hsa-mir-181b-1 // hsa-mir-181b-2 | 1.66E-04 | 1.947 |
| HBII-52-3 | 3.20E-04 | -1.264 |
| hsa-mir-103a-1 // hsa-mir-103a-2 | 3.68E-04 | 1.123 |
| HBII-13 | 4.07E-04 | 2.538 |
| hsa-mir-4521 | 6.14E-04 | 2.288 |
| hsa-mir-151 | 6.37E-04 | 1.376 |
| ENSG00000252657 | 7.34E-04 | -1.249 |
| hsa-mir-486 | 8.12E-04 | 3.509 |
| hsa-mir-4440 | 8.19E-04 | -1.768 |
| hsa-mir-4633 | 8.39E-04 | 1.308 |
| hsa-mir-1246 | 8.60E-04 | 5.001 |
| hsa-mir-630 | 9.82E-04 | 1.232 |
| ENSG00000251735 | 1.02E-03 | -1.278 |
| U20 | 1.44E-03 | 1.367 |
| HBII-336 | 1.56E-03 | 1.859 |
| U108 | 1.60E-03 | 1.472 |
| hsa-mir-4764 | 1.63E-03 | 1.249 |
| hsa-mir-4264 | 1.74E-03 | -1.181 |
| hsa-mir-1264 | 1.79E-03 | -1.239 |
| ENSG00000200693 | 1.79E-03 | -1.248 |
| hsa-mir-4444 | 1.81E-03 | 1.595 |
| mgU12-22-U4-8 | 1.81E-03 | 1.378 |
| ENSG00000207130 | 1.94E-03 | 1.837 |
| SNORD123 | 1.99E-03 | -1.290 |
| ENSG00000201410 | 2.09E-03 | -1.238 |
| ENSG00000252981 | 2.22E-03 | -1.104 |
| hsa-mir-513c | 2.38E-03 | -1.432 |
| ENSG00000239157 | 2.41E-03 | -1.268 |
| SNORD121B | 2.43E-03 | 1.429 |
| hsa-mir-891b | 2.45E-03 | -1.374 |
| hsa-mir-181a-1 | 2.58E-03 | 1.486 |
| ENSG00000252058 | 2.65E-03 | -1.259 |
| hsa-mir-194-1 // hsa-mir-194-2 | 2.84E-03 | 1.420 |
| ENSG00000239125 | 2.96E-03 | -1.188 |
| ENSG00000238929 | 3.07E-03 | 1.243 |
| ENSG00000238618 | 3.24E-03 | 1.098 |
| ENSG00000212249 | 3.49E-03 | 1.179 |
| hsa-mir-28 | 3.57E-03 | 1.863 |
| hsa-mir-4263 | 3.79E-03 | 1.361 |
| mgU6-77 | 3.97E-03 | 1.436 |
| hsa-mir-1825 | 4.38E-03 | -1.611 |
| hsa-mir-4282 | 4.52E-03 | 1.125 |
| U46 | 4.58E-03 | 2.144 |
| U101 | 4.62E-03 | 1.472 |
| ENSG00000252849 | 4.65E-03 | 1.165 |
| hsa-mir-181d | 4.78E-03 | 1.951 |
| U91 | 4.91E-03 | 1.422 |
| HBII-85-21 | 4.98E-03 | 1.481 |
| ENSG00000207118 | 5.03E-03 | 1.878 |
| mgh18S-121 | 5.08E-03 | 1.925 |
| HBII-251 | 5.18E-03 | 1.589 |
| HBII-13 | 5.20E-03 | 2.200 |
| U61 | 5.24E-03 | 1.362 |
| ENSG00000252040 | 5.25E-03 | -1.319 |
| ENSG00000207274 | 5.46E-03 | -1.222 |
| U72 | 5.50E-03 | 1.232 |
| U36A | 5.80E-03 | 1.368 |
| ENSG00000201807 | 6.00E-03 | -1.231 |
| ENSG00000212517 | 6.05E-03 | -1.234 |
| mgh28S-2409 | 6.25E-03 | 1.697 |
| ENSG00000238451 | 6.47E-03 | 1.176 |
| U63 | 6.47E-03 | 1.604 |
| SNORD121B | 6.87E-03 | 1.498 |
| ENSG00000201009 | 6.96E-03 | 1.901 |
| HBII-99 | 7.20E-03 | 1.359 |
| hsa-mir-4437 | 7.85E-03 | 1.150 |
| ACA5b | 7.88E-03 | 1.302 |
| hsa-mir-4740 | 7.92E-03 | -1.145 |
| hsa-mir-1281 | 8.33E-03 | -1.925 |
| HBII-210 | 8.44E-03 | 1.925 |
| hsa-mir-3192 | 8.89E-03 | -1.346 |
| hsa-mir-4800 | 9.15E-03 | 1.429 |
| HBII-85-12 | 9.23E-03 | -1.191 |
| U82 | 9.31E-03 | 1.619 |
| U71d | 9.66E-03 | 1.502 |
| ENSG00000207187 | 9.97E-03 | 1.735 |
