# Supplementary material for: Small ncRNA Expression-Profiling of Blood from Hemophilia A Patients Identifies miR-1246 as a Potential Regulator of Factor 8 Gene
Source: PLoS One. 2015 Jul 15;10(7):e0132433. doi: 10.1371/journal.pone.0132433 (PMC4503767; doi:10.1371/journal.pone.0132433)
Supplement: S5 File — (DOCX) [file pone.0132433.s005.docx]

**Supplemental Table 4.** Significantly differentially expressed ncRNAs between hemophilia A with- and without inhibitor development and controls identified by 3-class ANOVA (*P* value < 0.01).

| **Transcript ID** | ***P* value** | **Fold-Change (all HA vs. C)** | **Fold-Change (HAI vs. C)** | **Fold-Change (HAWI vs. C)** | **Fold-Change (HAI vs. HAWI)** |
| --- | --- | --- | --- | --- | --- |
| ENSG00000252834 | 3.48E-05 | 1.072 | 1.345 | -1.170 | 1.573 |
| hsa-mir-4437 | 8.63E-05 | 1.150 | 1.378 | -1.042 | 1.436 |
| hsa-mir-1246 | 8.98E-05 | 5.001 | 1.949 | 12.834 | -6.585 |
| ENSG00000238618 | 2.13E-04 | 1.098 | 1.029 | 1.173 | -1.141 |
| hsa-mir-597 | 2.85E-04 | -1.180 | -1.179 | -1.181 | 1.002 |
| hsa-mir-107 | 4.53E-04 | 1.130 | 1.156 | 1.105 | 1.047 |
| hsa-mir-181b-1 // hsa-mir-181b-2 | 6.18E-04 | 1.947 | 2.160 | 1.755 | 1.231 |
| HBII-13 | 7.66E-04 | 2.538 | 3.646 | 1.766 | 2.065 |
| ENSG00000200693 | 9.83E-04 | -1.248 | -1.433 | -1.088 | -1.317 |
| hsa-mir-655 | 1.00E-03 | 1.026 | 1.225 | -1.163 | 1.425 |
| hsa-mir-103a-1 // hsa-mir-103a-2 | 1.11E-03 | 1.123 | 1.158 | 1.089 | 1.064 |
| HBII-52-3 | 1.17E-03 | -1.264 | -1.308 | -1.220 | -1.072 |
| hsa-mir-151 | 1.21E-03 | 1.376 | 1.319 | 1.436 | -1.088 |
| hsa-mir-4444 | 1.34E-03 | 1.595 | 1.376 | 1.847 | -1.342 |
| hsa-mir-4633 | 1.45E-03 | 1.308 | 1.255 | 1.363 | -1.086 |
| ENSG00000252657 | 1.62E-03 | -1.249 | -1.222 | -1.276 | 1.044 |
| hsa-mir-4521 | 1.77E-03 | 2.288 | 2.885 | 1.814 | 1.591 |
| U23 | 1.86E-03 | 1.088 | 1.285 | -1.084 | 1.393 |
| hsa-mir-4740 | 1.97E-03 | -1.145 | -1.065 | -1.232 | 1.157 |
| hsa-mir-4440 | 1.98E-03 | -1.768 | -2.135 | -1.464 | -1.458 |
| ENSG00000238665 | 2.18E-03 | 1.033 | 1.240 | -1.163 | 1.442 |
| hsa-mir-630 | 2.46E-03 | 1.232 | 1.216 | 1.249 | -1.027 |
| hsa-mir-4263 | 2.52E-03 | 1.361 | 1.222 | 1.516 | -1.241 |
| ENSG00000251735 | 2.52E-03 | -1.278 | -1.385 | -1.180 | -1.174 |
| ENSG00000223111 | 2.54E-03 | -1.010 | 1.093 | -1.116 | 1.220 |
| hsa-mir-486 | 2.62E-03 | 3.509 | 3.620 | 3.400 | 1.065 |
| ENSG00000207109 | 2.98E-03 | -1.024 | 1.088 | -1.140 | 1.239 |
| ENSG00000201666 | 3.10E-03 | 1.086 | 1.212 | -1.028 | 1.246 |
| ENSG00000252981 | 3.59E-03 | -1.104 | -1.153 | -1.058 | -1.089 |
| HBII-85-12 | 3.65E-03 | -1.191 | -1.352 | -1.049 | -1.289 |
| HBII-336 | 3.70E-03 | 1.859 | 2.290 | 1.508 | 1.518 |
| mgU12-22-U4-8 | 4.41E-03 | 1.378 | 1.347 | 1.410 | -1.047 |
| hsa-mir-4764 | 4.57E-03 | 1.249 | 1.241 | 1.256 | -1.012 |
| U20 | 4.69E-03 | 1.367 | 1.383 | 1.350 | 1.025 |
| hsa-mir-4776-1 // hsa-mir-4776-2 | 4.79E-03 | -1.269 | -1.711 | 1.062 | -1.817 |
| hsa-mir-4264 | 4.82E-03 | -1.181 | -1.175 | -1.187 | 1.010 |
| hsa-mir-4282 | 5.17E-03 | 1.125 | 1.091 | 1.159 | -1.062 |
| U108 | 5.28E-03 | 1.472 | 1.592 | 1.361 | 1.169 |
| hsa-mir-3193 | 5.66E-03 | 1.164 | 1.483 | -1.094 | 1.622 |
| hsa-mir-1264 | 5.75E-03 | -1.239 | -1.298 | -1.182 | -1.098 |
| ENSG00000252058 | 5.82E-03 | -1.259 | -1.367 | -1.160 | -1.179 |
| ENSG00000201710 | 5.89E-03 | 1.087 | -1.037 | 1.226 | -1.271 |
| ENSG00000201410 | 5.96E-03 | -1.238 | -1.233 | -1.242 | 1.008 |
| hsa-mir-3136 | 6.07E-03 | 1.113 | -1.026 | 1.271 | -1.304 |
| hsa-mir-4703 | 6.15E-03 | -1.015 | 1.049 | -1.081 | 1.135 |
| ENSG00000212249 | 6.69E-03 | 1.179 | 1.152 | 1.206 | -1.046 |
| ENSG00000207130 | 6.69E-03 | 1.837 | 1.984 | 1.701 | 1.167 |
| SNORD123 | 6.84E-03 | -1.290 | -1.330 | -1.252 | -1.062 |
| hsa-mir-513c | 6.91E-03 | -1.432 | -1.575 | -1.302 | -1.210 |
| hsa-mir-4434 | 7.06E-03 | 1.088 | 1.200 | -1.013 | 1.216 |
| ENSG00000252849 | 7.22E-03 | 1.165 | 1.247 | 1.088 | 1.146 |
| HBII-13 | 7.23E-03 | 2.200 | 3.193 | 1.516 | 2.106 |
| hsa-mir-29b-1 | 7.37E-03 | -1.949 | -1.488 | -2.552 | 1.715 |
| SNORD121B | 7.61E-03 | 1.429 | 1.551 | 1.317 | 1.177 |
| ENSG00000239157 | 7.68E-03 | -1.268 | -1.335 | -1.204 | -1.108 |
| hsa-mir-891b | 7.76E-03 | -1.374 | -1.390 | -1.358 | -1.024 |
| ENSG00000238939 | 8.17E-03 | 1.168 | 1.357 | 1.005 | 1.350 |
| hsa-mir-3620 | 8.41E-03 | 2.019 | 1.489 | 2.739 | -1.839 |
| hsa-mir-181a-1 | 8.68E-03 | 1.486 | 1.541 | 1.432 | 1.077 |
| ENSG00000238654 | 8.71E-03 | -1.111 | 1.115 | -1.377 | 1.535 |
| hsa-mir-194-1 // hsa-mir-194-2 | 9.05E-03 | 1.420 | 1.530 | 1.318 | 1.161 |
| hsa-mir-25 | 9.29E-03 | 1.673 | 1.419 | 1.972 | -1.390 |
| ENSG00000239125 | 9.73E-03 | -1.188 | -1.226 | -1.151 | -1.065 |
| U80 | 9.73E-03 | 1.195 | 1.451 | -1.016 | 1.475 |
